# Perceived-air pollution and self-reported health status: a study on air pollution-prone urban area of Bangladesh

**DOI:** 10.3389/fpubh.2025.1382471

**Published:** 2025-04-03

**Authors:** Md Mostafizur Rahman, A. B. M. Hasanuzzaman, Musabber Ali Chisty, Edris Alam, Md Kamrul Islam, Abu Reza Md. Towfiqul Islam

**Affiliations:** ^1^Department of Disaster Management and Resilience, Faculty of Arts and Social Sciences, Bangladesh University of Professionals, Dhaka, Bangladesh; ^2^Institute of Disaster Management and Vulnerability Studies, University of Dhaka, Dhaka, Bangladesh; ^3^Department of Geography and Environmental Studies, University of Chittagong, Chittagong, Bangladesh; ^4^Faculty of Resilience, Rabdan Academy, Abu Dhabi, United Arab Emirates; ^5^Department of Civil and Environmental Engineering College of Engineering, King Faisal University, AlAhsa, Saudi Arabia; ^6^Department of Disaster Management, Begum Rokeya University, Rangpur, Bangladesh

**Keywords:** air pollution exposure, perception, self-reported health status, Dhaka city, Bangladesh

## Abstract

Air pollution is a serious health concern in rapidly developing countries like Bangladesh. This study investigates the correlation between self-reported health issues related to air pollution and perceived air pollution among adult Bangladeshis. A face-to-face questionnaire was conducted with 398 adult residents who had lived in their current location for at least 3 years. The survey assessed self-reported health using 13 specific air pollution-related health problems. A linear regression model was then used to analyze factors affecting air pollution-related health status. Our findings reveal correlations between perceived air pollution and health issues. 90% of respondents reported air-polluted environments in their area, with the majority citing multiple sources (42%) of air pollution. Construction activities emerged as a predominant concern, identified by 31% of participants as a primary source of air pollution. Demographic factors play a crucial role in contributing to air pollution-related health problems. Notably, older age groups reported significantly higher health issues compared to their younger counterparts. Residents of Mirpur’s residential neighborhood experienced fewer health problems related to air pollution, indicating the influence of urban planning on public health. This interdisciplinary approach offers a comprehensive view of Bangladesh’s air pollution crisis, combining environmental science and public health perspectives. The findings emphasize the need for targeted policy interventions, including stricter regulations on construction activities to mitigate their impact on air quality, tailored public health interventions for vulnerable populations (especially older adults), and urban planning strategies that reduce exposure to air pollution in residential areas. Future research should investigate the long-term health impacts of chronic air pollution exposure and evaluate the effectiveness of various mitigation strategies. Addressing these issues can help create healthier, more resilient urban environments.

## 1 Introduction

Air pollution is a significant global concern, the largest environmental danger to human health, and the cause of one out of every nine annual fatalities (1). The world’s largest health burden comes from air pollution (2, 3). Gaseous and solid particles, including indoor and outdoor air pollution, cause air pollution. Outdoor air pollution, the “modern form of pollution,” has caused more deaths and disabilities than interior air pollution worldwide (4, 5). This invisible threat knows no borders, affecting developed and developing nations. It has emerged as one of our most pressing environmental challenges, casting a long shadow over public health and economic development worldwide. 99% of the global population breathes air that exceeds WHO guideline limits, containing high levels of pollutants (6). In 2019, ambient air pollution killed 4.5 million people, roughly twice as much indoor air pollution, especially in South Asia, East Asia, and Southeast Asia (4). In the last decade, research has increasingly highlighted the pervasive nature of air pollution. A study estimated that outdoor air pollution leads to 3.3 million premature deaths per year globally, with the majority occurring in Asia (7). This statistic underscores the urgency of addressing air quality on a global scale. Climate change will affect air quality in highly populated areas through atmospheric ventilation, dilution, removal processes, and precipitation changes, worsening the situation (8). Urban air pollution is a major issue since pollutant concentrations and potential victims are concentrated (9–11). Rapid urbanization, particularly in developing countries, has exacerbated air pollution concerns. The urban landscape can trap pollutants, creating “urban heat islands” that concentrate harmful substances. Poor urban air quality is becoming a major issue as more people relocate to cities and demand a clean, healthy atmosphere (12). Projected global population expansion, urbanization, and climate change influences on air conditions and weather variability exacerbate this (1, 13). Urbanization is a key indicator of economic growth. Urbanization helps human growth because urban inhabitants enjoy greater urban facilities, amenities, and public services; thus, they relocate to urban regions for jobs and better amenities, expanding urban areas (14). Significant evidence shows that airborne particles in this area harm health (15). According to studies, this is caused by increased private automobiles, unmaintained vehicles, low-quality gasoline, badly paved roads, and improper urban infrastructural activities (16). Thus, industrialization-caused air pollution is among the 10 biggest environmental health hazards associated with global mortality (17, 18). Research demonstrated how urban planning and design could either mitigate or exacerbate air pollution, highlighting the need for sustainable urban development strategies (19).

With its dense population and rapid industrial growth, Bangladesh faces particularly acute air quality issues. The Air Quality Life Index (AQLI) ranks Bangladesh as the world’s most air-polluted country, and it has highlighted four densely populated areas and the capital city, Dhaka, as the most vulnerable to air pollution-induced death loss (20, 21). Despite a 2.1% drop in particulate pollution from 2020, Bangladesh has had pollution 14 to 15 times the WHO standard for a decade. According to the index, particulate pollution reduces life expectancy by 6.8 years in Bangladesh, second only to cardiovascular disorders (20). Bangladesh’s air pollution sources are diverse, ranging from brick kilns and vehicle emissions to construction dust and industrial processes. For example, coal-burning brick kilns were found to be a major local air pollution source in Dhaka (22), with some 1,000 brick kilns spread across the Dhaka metro region (23). Still, these kilns only operate only during the dry seasons (24). Environmental stressors have been linked to several diseases. Children, women, and older people are especially sensitive to respiratory problems (25). It also contains chest infections, which can lead to influenza, pneumonia, lung cancer, etc. (26). People exposed to high pollution levels are prone to these issues. A cohort study found a relationship between exposure to high air pollutants and increased incidence of chronic obstructive pulmonary disease (COPD) in urban Bangladesh (27). Research demonstrated a significant association between short-term exposure to air pollution and hospital admissions for cardiovascular diseases in Dhaka (28). Air pollution is becoming the biggest worry, especially in Dhaka. This mega city has consistently ranked among the world’s most polluted cities in recent years. Dhaka is the country’s center in every way; every day, people come to this metropolis for jobs, treatment, and education. They are putting the air pollution issue beyond management. A study found that PM2.5 levels in Dhaka often exceed WHO guidelines by 5–10 times during the dry season (29).

Air pollution can harm physical and mental health, emphasizing the need for public health initiatives and effective communication (30, 31). Air pollution perception influences mobility, health, well-being, and quality of life (32, 33). Moreover, air quality perception might trigger psychological suffering (34). Since exposure to air pollution can lower life satisfaction and happiness, overall quality of life is impacted.

People’s perceptions of air quality play a crucial role in responding to health risks. The perceived air pollution has been shown in several studies (35–40). Observed air pollution measurements can be complemented by perceptions of air pollution (41). According to research, people’s perceptions of air quality affect how they perceive and act on health risks (41). If there were increased levels of air pollution, exposure may have directly impacted how polluting things were perceived, how health risks were regarded, symptoms, or illnesses. For instance, compared to the Respiratory Health in Northern Europe (RHINE) cohort, which was exposed to substantially lower quantities of air pollution, researchers found a far greater association between simulated air pollution and self-reported traffic intensity in Rome (42). However, a global study found that individuals often underestimate air pollution levels in their local area (43). A more thorough knowledge of the impact of air quality on public health is possible by combining perceived exposure with objective measures (44). This method helps determine how well air quality control plans and regulations work. Studies examining the relationship between pollution exposure and air quality perceptions help policymakers decide how to improve air quality and public health (45). In general, integrating both observable data and perceived air pollution helps provide a more complete picture of the effects of air pollution on human health and guides successful responses. It is crucial to consider how it is perceived to evaluate air pollution and its effects on the population, particularly in polluted areas.

Understanding how people perceive air quality requires robust research methodologies. Large-scale surveys remain a cornerstone of perception research. A study assessed air quality perceptions and reported behaviors among four Spanish cities (46). They focused on how different cities’ populations perceive air pollution, risk perceptions, and self-reported mitigation measures for ambient air pollution. Another study was on public awareness of air pollution and health threats (47). Statistical analyses of datasets can reveal patterns in perception. A multi-divisional study assessed the perception of air pollution in Bangladesh (48). Self-reported health includes psychological, social, and physical health that cannot be measured by morbidity (49, 50). Low self-reported health is strongly linked to early death because it is intrinsically tied to this larger picture of health (49). Self-reported health surveys may show a range of health statuses due to social, economic, and environmental variables. In most cases, self-reported health surveys ask, “How would you evaluate your general health…” followed by three to five ratings (poor, medium, fair health condition). Social class and environmental issues including air, noise, and waste pollution may affect health. The combination of air pollution and weather events, like heat waves and cold spells, might impact self-reported health assessments by affecting the body’s thermoregulation system (51–53). It is commonly acknowledged that extreme weather occurrences inherently worsen the impact of air pollution. In particular, heat waves and excessive temperatures during them harm human health, raising the risk of respiratory illness and mortality (52).

Self-reported health status serves as a crucial indicator of population health and well-being. In the context of air pollution, it can provide valuable insights into how people perceive the impact of environmental factors on their health. The relationship between perceived air pollution and self-reported health status is complex and multifaceted. It highlights the critical need for further research into the interplay between air pollution, public perception, and health outcomes, particularly in rapidly developing urban areas like Bangladesh. However, a lack of study exists on the relationship between air pollution and self-reported health status in Bangladesh. By addressing it, we can develop more effective strategies for communicating air pollution risks, promoting protective behaviors, and improving public health in this global environmental challenge.

This research aims to understand how perceived air pollution affects self-reported health in adult Bangladeshis. We evaluated various air pollution-related health concerns from past research when assessing self-reported health status (6, 54–58). Several Bangladeshi air pollution studies have been done (25, 26, 59, 60). However, this is the first study to examine how Bangladesh’s adult population perceives air pollution and self-reports air pollution-related health issues. The findings of this study might facilitate the development of regional and local air pollution mitigation plans.

## 2 Research methodology

### 2.1 Research design

A self-reported health survey was developed to evaluate how respondents perceived health issues connected to air pollution. This study continued the earlier one in which we assessed the health issues caused by noise in Dhaka (61). The boundary conditions were age (18 years and older) and residency for at least 3 years in our study areas.

### 2.2 Study area

**Figure 1:**
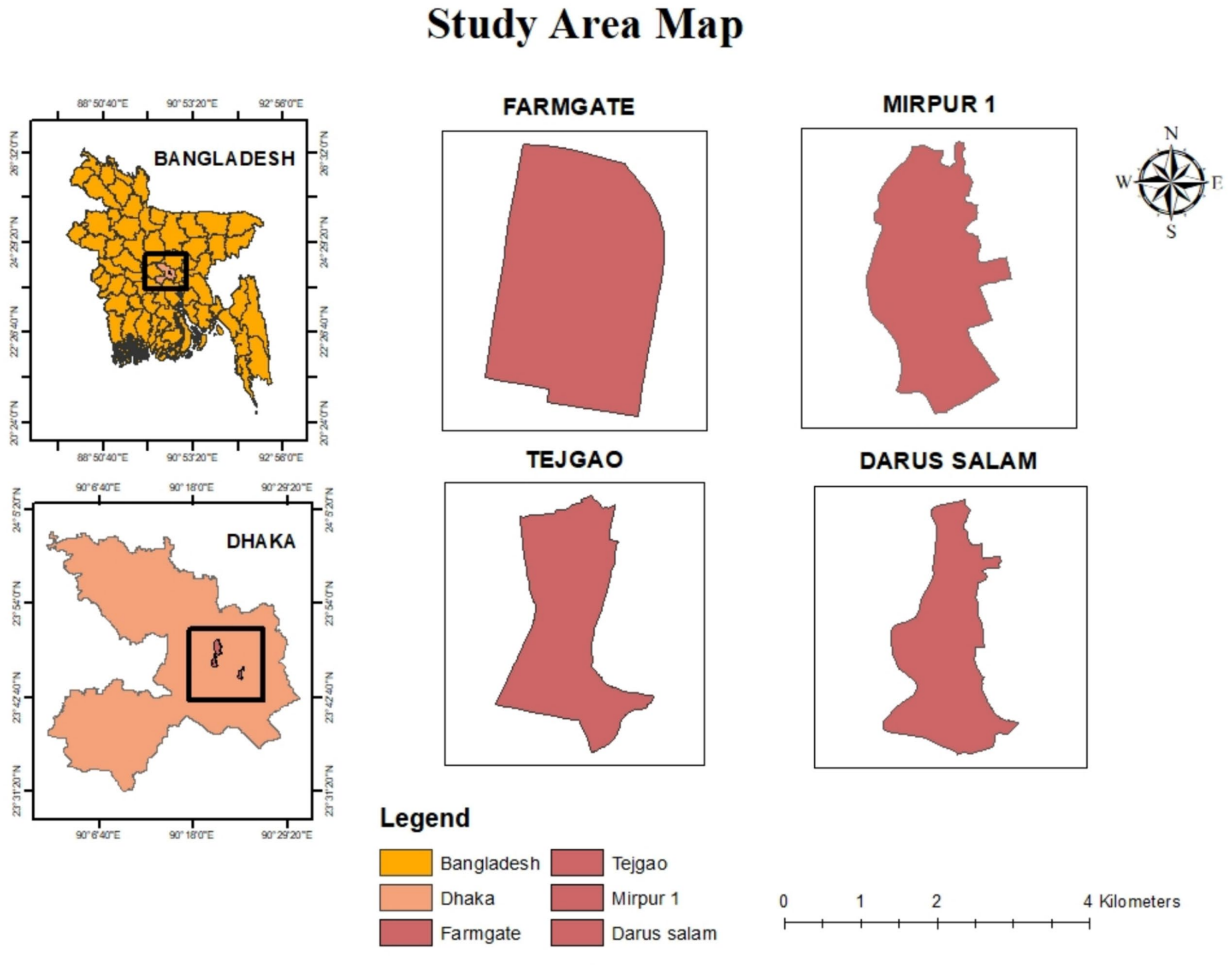
The study area.

As we have already discussed, Dhaka is one of the cities with the worst air quality. Tejgaon, Darus Salam, Mirpur-1, and Farmgate were the research areas in Dhaka (Figure 1). Due to the industrial activity and heavy traffic, the air in Tejgaon, Darus Salam, and Farmgate neighborhoods appeared polluted. However, Mirpur-1 was chosen as a residential neighborhood.

Figure 1 is being embedded below here.

### 2.3 Survey tool

The questionnaire’s draft was prepared by following earlier research (6, 54–58). Before developing the final questionnaire, a pilot survey was also carried out, but it was excluded from the final analysis. Four elements made up the final questionnaire (available in both local Bangla and English): sociodemographic data, perceived local air pollution, individual perspectives on air pollution, and the health issues associated with air pollution. In the last phase, participants self-reported their health state. They were asked to “please respond to the following health concerns compared to 3–5 years ago” concerning any health disorders connected to air pollution. It listed 13 health issues caused by air pollution, including shortness of breath (especially when exercising), breathing difficulty even when not exercising, respiratory tract irritation and inflammation (coughing), chest tightness, pain when inhaling deeply, sinus issues, heart disease, lung cancer, aggravated asthma, type 2 diabetes, systemic inflammation, dementia, and wheezing and coughing (6, 54–58) with “About the same/Better/Worst” option. A score of one was given for a positive answer, while a score of 0.50 and 0 were given for a neutral and negative response, respectively.

### 2.4 Sampling

Between mid-July and mid-August 2022, a face-to-face survey was conducted. The questionnaire was made using Google Forms. The sample strategy used in this investigation was non-probability convenience sampling. People living in our selected areas were selected based on our convenience to contact. For this perception-based study, Morgan’s Table indicated that a minimum sample size of 384 people (95% Confidence Interval) was necessary (62). We successfully reached 412 respondents. After data processing, however, the final survey comprised 398 participants.

### 2.5 Data analysis

Python (version 2.7; Beaverton, OR 97008, USA) and RStudio (version 1.2.5042; Boston, MA, USA) (63, 64) were utilized for data administration and analysis. All statistical analyses were conducted using a 95% confidence interval. When relevant, descriptive analyses (frequency and percentage) were calculated. Like previous studies for noise pollution (61), the average of the 13 scores for health issues linked to air pollution was used to generate the overall score for health problems connected to air pollution. We have examined the factors of self-reported total air pollution-related health concerns with linear regression. We have undertaken further multiple linear regression analysis using sociodemographic factors of significance (in linear regression analysis).

### 2.6 Ethical issue

This research has been approved by the Disaster Management & Resilience department, Bangladesh University of Professionals, Dhaka, Bangladesh. This study is part of approved research from the Department of Disaster Management & Resilience, Bangladesh University of Professionals, Dhaka, Bangladesh. In addition, it was approved by the Institute of Disaster Management and Vulnerability Studies research ethics review committee (ERC-02/02/2021), University of Dhaka, Bangladesh. Following the human subject, it has maintained all related ethical concerns. Consent was taken. No incentives were given to take the survey.

## 3 Results

### 3.1 Air pollution-related health problem by sociodemographic profile

The total health issue brought on by air pollution is segmented by sociodemographic data in Table 1. Many people we studied were between 36 and 45, 18 and 25, and 46 and 55. Participants were male at 67% and female at around 33%. Most of them were unmarried and were living with their families. Both high-rise and low-rise buildings housed a large number of people. Many claimed to have a job and less than a tertiary degree in employment and education level, respectively.

**Table 1:** Overall health issue resulting from air pollution by sociodemographic profile (*n* = 398).

Features	Overall air pollution-related health problem			
	*n* (%)	R^2^	B^#^	*p*-value
1. Age (Year)				
18–25	100 (25.13)		Reference	
26–35	81 (20.35)		0.01	0.390
36–45	104 (26.13)	0.016	0.03	0.073
46–55	88 (22.11)		0.05^**^	0.005
>55	25 (6.28)		0.06^*^	0.040
2. Gender				
Female	133 (33.42)		Reference	
Male	265 (66.58)	0.001	−0.01	0.444
3. Marital status				
Married	289 (72.61)		Reference	
Unmarried	109 (27.39)	0.012	−0.03^*^	0.030
4. Living with family				
No	89 (22.36)		Reference	
Yes	309 (77.64)	0.000	−0.00	0.891
5. Location				
Darus Salam	97 (24.37)	0.073	Reference	
Farmgate	95 (23.87)		0.03	0.060
Mirpur	99 (24.87)		−0.05^**^	0.001
Tejgaon	107 (26.88)		0.02	0.198
6. Residence				
High-rise building^a^	149 (37.44)	0.012	−0.03	0.057
Low-rise building^b^	139 (34.92)		−0.01	0.487
Mixed-use building^c^	68 (17.09)		Reference	
Slums	42 (10.55)		−0.03	0.13
7. Occupation				
Employed	189 (47.49)	0.030	0.02	0.216
Unemployed	104 (26.13)		0.02	0.424
Day labour	43 (10.80)		Reference	
Students	62 (15.58)		−0.03	0.150
8. Education level				
Tertiary	167 (41.96)	0.007	Reference	
<Tertiary	203 (51.01)		−0.02	0.102
No education	28 (7.04)		−0.01	0.598

The results of a multiple linear regression analysis are shown in Table 2. Mirpur’s residential neighborhood participants exhibit fewer symptoms of air pollution-related illness.

# Beta.

* *p < 0.05.*

** *p* < 0.01. ***p < 0.001.

a High-rise building = > 5 storey.

b Low-rise building = <= 5 storey.

c Mixed-use building = accommodation, shops, market etc. together.

When compared to the youngest age group (18–25 years), the older age groups (46–55 and >55 years) reported a considerably higher number of health issues connected to air pollution. Regarding marital status, those who were unmarried reported much less hardships. Residents of Mirpur’s residential neighborhood had noticeably fewer health issues caused by air pollution (Table 2).

**Table 2:** Overall health issue resulting from air pollution by sociodemographic profile (multiple linear regression analysis).

Features	Overall air pollution-related health problem		
	R^2^	B^#^	*p*-value
1. Age (Year)	0.097		
18–25			
26–35		0.01	0.446
36–45		0.02	0.424
46–55		0.04	0.065
>55		0.05	0.104
2. Marital status			
Married			
Unmarried		−0.01	0.535
3. Location			
Darus Salam			
Farmgate		0.03	0.111
Mirpur		−0.06^***^	0.000
Tejgaon		0.02	0.222

# Beta.

* *p < 0.05.*

** *p < 0.01.*

*** *p < 0.001.*

### 3.2 Air pollution in the locality

Appendix Table 1 shows that most participants (90%) reported air pollution environments in the neighborhood from various sources. The majority of them (42%) reported mixed kinds, followed by construction activities (31%) and all sorts of air pollution (30%) prevalent in the Dhaka city. Many people had the belief that local air pollution could be reduced. About 57% of respondents say the monsoon season has the least air pollution. About half of people thought that indoor air pollution existed.

People who stated that their local area had high levels of air pollution due to construction and the combustion of fossil fuels showed substantial health problems. Participants who believed air pollution could not be controlled showed high suffering. The number of health issues caused by air pollution was much higher among people who identified winter as the most polluted season. According to Appendix Table 2, 55% of the participants in the survey thought that air pollution was responsible for illnesses in their friends and family. Additionally, they believed that air pollution was harmful to health. Many wanted to participate in campaigns to reduce air pollution but were unaware of them. More than 80% of respondents said there should be more information accessible about air pollution. According to about 59% of respondents, only government and non-government groups should not be in charge of limiting air pollution; however, they also think family and friends may help. About 80% concurred that taking precautions against air pollution might lessen health risks. However, many of them either never used masks or did not wear them whenever they went outside. Many also consented to paying higher taxes and other fees to lower air pollution. However, almost 55% did not notice the daily weather prediction before heading outside.

Participants unwilling to engage in an awareness campaign on air pollution experienced intense suffering. Participants responded that no further information should be made available to the general public regarding information relating to air pollution, which reported noticeably high suffering. Less suffering was seen in respondents who did not think that only governments and non-governmental organizations should be in charge of reducing air pollution. However, those who did not think that friends and family might help minimize air pollution reported suffering considerably higher rates. Respondents who thought taking precautions against air pollution is crucial to lowering health risks reported less suffering. Similarly, less suffering was reported when wearing a mask and going outside.

## 4 Discussion

We evaluated the association between self-reported air pollution-related health issues, sociodemographic data, and perceived air pollution in the locality. We noticed that participants’ age group, marital status, and location were all significant factors in air pollution-related health concerns. Earlier studies revealed an association between sociodemographic status and perceived air pollution exposure (37, 65, 66). Several studies examined sociodemographic data to assess self-reported air pollution-related health status (67–69). Similarly, we assessed self-reported noise-related health status based on sociodemographic data (61). We observed that older people reported air pollution-related health concerns, supporting another study that suggested older people may be more sensitive to air pollution (70–72). Air pollution harms older individuals since they often have many chronic illnesses, including high blood pressure, diabetes, and heart disease. Older adults are more likely to be hospitalized when air pollution is excessive. Ageing causes a gradual reduction in physical function, making aged people more vulnerable, fragile, and sensitive. The immune system changes with age, reducing its ability to react to infection and immunization and increasing infectious disease morbidity and mortality in older people (73).

Residential respondents reported less air-pollution-related suffering than industrial and traffic respondents. Residential, industrial, and transportation air pollution levels vary greatly (74–76). Due to industrial, car, and construction pollutants, industrial and traffic regions have greater air pollution levels (74). Residential pollution is lower, although cooking and heating can contribute. Studies reveal that industrial and transportation regions have greater PM, NO_2_, and SO_2_ levels than residential areas (74). Air quality monitoring is essential to identify health hazards and minimize pollution. Living near roads and large roadways can significantly increase pollution, stressing the significance of air quality in urban development.

The effectiveness of any policy to control pollutant emissions involves public acceptance that, in turn, necessitates a proper understanding of the primary pollutant emission drivers (39). According to our research, construction is the main source of air pollution. Air pollution is largely caused by construction (77–79). They discharge air pollutants into the atmosphere, including dust, particulate matter, diesel exhaust, and volatile organic compounds (VOCs) (80–82). Heavy machinery, diesel engines, land clearance, demolition, and rubbish burning cause construction-related air pollution. According to studies, construction materials make up nearly 38% of outdoor air pollution (83). Air quality and public health are affected by these contaminants. Cleaner machinery, dust management, and sustainable building methods reduce construction pollution (84, 85). Construction air pollution must be reduced to safeguard the environment and adjacent residents. Air pollution in Dhaka is largely caused by construction (86).

Rapid urbanization, construction, road digging, and industrial growth in Dhaka add to the issue. The Air Quality Research and Monitoring Centre of Dhaka University’s chemistry department said that dust and smoke cause 50% of the city’s pollution (87). In addition, it has been claimed that mismanaged construction and ageing car exhausts cause this dust. Effective pollution control, sustainable building, and government involvement are needed to reduce construction-related air pollution’s negative impacts on the city’s environment and public health (87). Following a written appeal against air pollution in Dhaka, the High Court issued nine instructions to the Department of Environment and the two municipal corporations in January 2020 (87). The directives included covering trucks and other vehicles carrying sand, soil, or garbage in Dhaka city; covering soil, sand, cement, and stones on construction sites; spraying streets regularly; following tender conditions for road, culvert, carpeting, and digging; and enforcing road transport act, time limits for vehicle movement (87).

This study also examined air pollution attitudes. People with more serious air pollution-related illnesses did not believe their location could manage air pollution. Due to a sickness’s physical and psychological effects, one may lose faith in their potential to heal (88, 89). Diseases can lower self-esteem, making people doubt their skills. Chronic disease can reduce self-esteem and self-efficacy. These encounters can cause anxiety and despair. Negative emotions might hamper rehabilitation, so confront them and get help. Recognizing how the condition affects self-perception and engaging in self-esteem and well-being might help rebuild confidence (89). Many respondents said that winter had the least air pollution, consistent with a prior study at the US embassy in Dhaka (60). They found that winter air pollution is higher and monsoon pollution is lowest.

We observed that few respondents knew about air pollution-related campaigns, but many were interested. Campaigns against air pollution are vital to increasing awareness and promoting healthier air. These initiatives, generally launched by groups, governments, and health authorities, educate the public about air pollution’s health consequences and encourage action. The Environment & Clean Air Coalition and WHO support the BreatheLife Campaign, which encourages cities and individuals to cut air pollution for better health and a safer climate (90). The UK Clean Air Day promotes air pollution reduction (91). Air pollution affects health and the environment; thus, campaigns provide information, tactics, and policies. Such actions are essential for a shared effort to clean the air and improve health. Increasing public air pollution knowledge is also important. Air pollution causes many diseases and deaths globally. This issue needs public knowledge and understanding (92). Accessible information helps people understand air pollution, its causes, health effects, and solutions. Anthropogenic environmental contamination is tough to eliminate, but a close partnership between authorities, bodies, and healthcare providers might fix it. Governments should educate people and involve specialists to control the problem’s rise (92). Informative material inspires individuals to decrease pollution, advocate for cleaner air regulations, and act. By making such information available, we can improve air quality, public health, and the environment. Our study population thought they should minimize air pollution, not only governments or NGOs. Our participants did not always wear masks outside, where air pollution is prevalent. Participants using masks suffered less from air pollution. Wearing masks can reduce air pollution and its health impacts (93). N95 respirators and pollution masks protect against airborne particulates and contaminants to varied degrees (94). Masks filter out particles and contaminants, especially during heavy air pollution. According to research, masks help reduce PM2.5 exposure, especially in metropolitan locations with strong traffic emissions (93). However, mask selection and fit affect effectiveness. Masks can reduce air pollution’s health risks but should be used with other interventions.

Air pollution reduction requires a comprehensive approach. The Ministry of Environment, Forest and Climate Change has released the Air Pollution (Control) Rules (APCR), 2022, as a gazette notice, intending to combat the severe air pollution in Bangladesh (95). However, the policy must be monitored and implemented. The authority should work with central government, local governments, educational institutions, private offices, health professionals, environmental experts, disaster management practitioners, and community leaders to spread knowledge, promote air pollution awareness, and implement preventive measures. Along with frequent air pollution inspections, governments must organize campaigns, social mobilization, and communication about air pollution management techniques to educate and teach communities to tackle this major public health issue. Television and social media may broadcast educational projects like short films and case studies regarding community-level air pollution management. Social media has become a major source of information for Bangladeshis (96). These methods must incorporate literacy and cognitive comprehension while designing and implementing them (97). The authorities must equip and train its staff and other stakeholders to mitigate this major public health concern. To create effective air pollution mitigation methods, authorities, researchers, companies, and communities must work together. The government should fine air polluters more. They may also perform several studies to determine new air pollution remedies. Beijing, infamous for its air pollution, has maintained stable GDP growth for the previous three decades despite investing in and controlling air quality improvement projects, which have improved air quality by 50% (95, 98). It was achieved through cost-effective planning and determined execution. In addition, several studies have recommended that urban vegetation reduce air pollution (99, 100).

## 5 Limitations

The study has some limitations that should be noted when evaluating the results. We used self-reported health assessments of Bangladesh’s adult population, not clinical diagnoses. Thus, study findings should be viewed carefully. Second, it employed non-probability sampling with a questionnaire survey that may be biased. Even if the survey pattern is anonymous, respondents may consider socially acceptable responses. Third, it only measured perceived air pollution, which may not reflect pollution levels. However, this exploratory study may help Bangladeshi authorities and other affected people reduce air pollution.

## 6 Conclusion

It is the first research in Bangladesh to examine how individuals perceive their health concerning air pollution. The busy streets of Dhaka, once filled with the vibrant energy of millions, now bear witness to an invisible threat that looms over its residents. Our study unveils a reality: air pollution is not just an environmental concern but a pressing public health crisis that demands immediate attention. Our findings show a picture of vulnerability increasing with age. The older generations, who have witnessed Dhaka’s rapid transformation, now face the harshest consequences of its progress. The age groups 46–55 and above 55 reported significantly higher health issues related to air pollution than their younger counterparts. This disparity calls for targeted interventions to protect our elders, the bearers of our cultural wisdom. The study reveals a cruel irony: winter, traditionally a season of joy and festivities in Bangladesh, emerges as the most polluted period. The air that should bring relief instead carries a higher burden of pollutants, exacerbating health issues. Conversely, the monsoon season offers a brief respite, with lower pollution levels reported. This seasonal variation underscores the need for dynamic, season-specific strategies to combat air pollution. Most participants recognize the health hazards of air pollution and express a willingness to take action. However, a critical gap exists between awareness and action. Despite understanding the risks, many residents do not consistently use protective measures like masks. This disconnect highlights the urgent need for comprehensive public education campaigns that inform and motivate behavioral changes. Our study confirms that simple actions can make a significant difference. Participants who regularly wore masks when outdoors reported fewer health issues. This finding emphasizes the importance of promoting and facilitating protective gear as a defense against air pollution. The path forward is clear yet challenging. We must implement stringent air quality regulations to curb pollution at its source. We need to launch targeted public health initiatives focusing on vulnerable age groups. Authority must develop urban planning strategies prioritizing green spaces and sustainable transportation. We should create community engagement programs to bridge awareness and action gaps. While our study sheds light on the current situation, it also illuminates paths for future research. We need to delve deeper into the long-term health effects of chronic exposure to air pollution, the economic impacts of pollution-related health issues on Dhaka’s economy, innovative technological solutions for air quality improvement, and comparative studies across major South Asian cities to identify best practices.

## Data Availability

The raw data supporting the conclusions of this article will be made available by the authors, without undue reservation.

## References

[1] World Health Organization. (2016). Ambient air pollution: a global assessment of exposure and burden of disease. World Health Organization. p. 121. Available online at: https://apps.who.int/iris/handle/10665/250141 (Accessed August 29, 2023).

[2] Hänninen O Knol AB Jantunen M Lim T-A Conrad A Rappolder M. Environmental burden of disease in Europe: assessing nine risk factors in six countries. Environ Health Perspect. (2014) 122:439–46. doi: 10.1289/ehp.1206154, PMID: 24584099 PMC4014759

[3] Clark C Paunovic K. WHO environmental noise guidelines for the European region: a systematic review on environmental noise and quality of life, wellbeing and mental Health. Int J Environ Res Public Health. (2018) 15:2400. doi: 10.3390/ijerph15112400, PMID: 30380665 PMC6266190

[4] Fuller R Landrigan PJ Balakrishnan K Bathan G Bose-O’Reilly S Brauer M. Pollution and health: a progress update. Lancet Planet Health. (2022) 6:e535–47. doi: 10.1016/S2542-5196(22)00090-0, PMID: 35594895 PMC11995256

[5] Institute HE (2019). State of global air 2019. Spec Rep.

[6] WHO (2023) Air pollution. Available online at: https://www.who.int/health-topics/air-pollution (Accessed August 22, 2023).

[7] Lelieveld J Evans JS Fnais M Giannadaki D Pozzer A. The contribution of outdoor air pollution sources to premature mortality on a global scale. Nature. (2015) 525:367–71. doi: 10.1038/nature15371, PMID: 26381985

[8] Fiore AM Naik V Leibensperger EM. Air quality and climate connections. J Air Waste Manage Assoc. (2015) 65:645–85. doi: 10.1080/10962247.2015.1040526, PMID: 25976481

[9] Kumar P Khare M Harrison RM Bloss WJ Lewis AC Coe H. New directions: air pollution challenges for developing megacities like Delhi. Atmos Environ. (2015) 122:657–61. doi: 10.1016/j.atmosenv.2015.10.032

[10] Mukherjee A Agrawal M. World air particulate matter: sources, distribution and health effects. Environ Chem Lett. (2017) 15:283–309. doi: 10.1007/s10311-017-0611-9

[11] Kumar P de Fatima AM Ynoue R Fornaro A Dias de Freitas E Martins J. New directions: from biofuels to wood stoves: the modern and ancient air quality challenges in the megacity of Sao Paulo. Atmos Environ. (2016) 140:364–9. doi: 10.1016/j.atmosenv.2016.05.059

[12] Sicard P Agathokleous E Anenberg SC De Marco A Paoletti E Calatayud V. Trends in urban air pollution over the last two decades: a global perspective. Sci Total Environ. (2023) 858:160064. doi: 10.1016/j.scitotenv.2022.160064, PMID: 36356738

[13] Tibbetts JH. Air quality and climate change: a delicate balance. Environ Health Perspect. (2015) 123:A148–53. doi: 10.1289/ehp.123-A148, PMID: 26030069 PMC4455574

[14] Arfanuzzaman M Dahiya B. Sustainable urbanization in Southeast Asia and beyond: challenges of population growth, land use change, and environmental health. Growth Chang. (2019) 50:725–44. doi: 10.1111/grow.12297

[15] Flood-Garibay JA Angulo-Molina A Méndez-Rojas MÁ. Particulate matter and ultrafine particles in urban air pollution and their effect on the nervous system. Environ Sci Process Impacts. (2023) 25:704–26. doi: 10.1039/D2EM00276K, PMID: 36752881

[16] Taghizadeh F Mokhtarani B Rahmanian N. Air pollution in Iran: the current status and potential solutions. Environ Monit Assess. (2023) 195:737. doi: 10.1007/s10661-023-11296-5, PMID: 37233853 PMC10213600

[17] Singh R Singh V Gautam AS Gautam S Sharma M Soni PS. Temporal and spatial variations of satellite-based aerosol optical depths, angstrom exponent, single scattering albedo, and ultraviolet-aerosol index over five polluted and less-polluted cities of northern India: impact of urbanization and climate change. Aerosol Sci Eng. (2023) 7:131–49. doi: 10.1007/s41810-022-00168-z

[18] Adami G Pontalti M Cattani G Rossini M Viapiana O Orsolini G. Association between long-term exposure to air pollution and immune-mediated diseases: a population-based cohort study. RMD Open. (2022) 8:e002055. doi: 10.1136/rmdopen-2021-002055, PMID: 35292563 PMC8969049

[19] Grimmond S Bouchet V Molina LT Baklanov A Tan J Schlünzen KH. Integrated urban hydrometeorological, climate and environmental services: concept, methodology and key messages. Urban Clim. (2020) 33:100623. doi: 10.1016/j.uclim.2020.100623, PMID: 32292692 PMC7128437

[20] Tajmim T Rahman S. (2023) Air pollution keeps shortening Bangladeshi people’s lives as density grows: study. Bus Stand. Available online at: https://www.tbsnews.net/bangladesh/health/air-pollution-keeps-shortening-bangladeshi-peoples-lives-density-grows-study (Accessed August 30, 2023).

[21] The Air Quality Life Index (AQLI). (n.d.). AQLI. Available online at: https://aqli.epic.uchicago.edu/the-index/ (Accessed August 30, 2023).

[22] Begum BA Hopke PK. Identification of sources from chemical characterization of fine particulate matter and assessment of ambient air quality in Dhaka, Bangladesh. Aerosol Air Qual Res. (2019) 19:118–28. doi: 10.4209/aaqr.2017.12.0604

[23] Guttikunda SK Kopakka RV. Source emissions and health impacts of urban air pollution in Hyderabad, India. Air Qual Atmos Health. (2014) 7:195–207. doi: 10.1007/s11869-013-0221-z

[24] Rahman MM Begum BA Hopke PK Nahar K Thurston GD. Assessing the PM2.5 impact of biomass combustion in megacity Dhaka, Bangladesh. Environ Pollut. (2020) 264:114798. doi: 10.1016/j.envpol.2020.114798, PMID: 32559884 PMC9581344

[25] Begum BA Biswas SK Markwitz A Hopke PK. Identification of sources of fine and coarse particulate matter in Dhaka, Bangladesh. Aerosol Air Qual Res. (2010) 10:345–53. doi: 10.4209/aaqr.2009.12.0082

[26] Begum BA Biswas SK Hopke PK. Impact of banning of two-stroke engines on airborne particulate matter concentrations in Dhaka, Bangladesh. J Air Waste Manage Assoc. (2006) 56:85–9. doi: 10.1080/10473289.2006.10464430, PMID: 16499150

[27] Alam DS Chowdhury MA Siddiquee AT Ahmed S Clemens JD. Prevalence and determinants of chronic obstructive pulmonary disease (COPD) in Bangladesh. COPD J Chronic Obstr Pulm Dis. (2015) 12:658–67. doi: 10.3109/15412555.2015.1041101, PMID: 26263031 PMC9747524

[28] Khan R Konishi S Ng CFS Umezaki M Kabir AF Tasmin S. Association between short-term exposure to fine particulate matter and daily emergency room visits at a cardiovascular hospital in Dhaka, Bangladesh. Sci Total Environ. (2019) 646:1030–6. doi: 10.1016/j.scitotenv.2018.07.288, PMID: 30235588

[29] Begum BA Biswas SK Hopke PK. Key issues in controlling air pollutants in Dhaka, Bangladesh. Atmos Environ. (2011) 45:7705–13. doi: 10.1016/j.atmosenv.2010.10.022

[30] WHO (2023). How air pollution is destroying our health. Available online at: https://www.who.int/news-room/spotlight/how-air-pollution-is-destroying-our-health (Accessed August 30, 2023).

[31] Riley R de Preux L Capella P Mejia C Kajikawa Y de Nazelle A. How do we effectively communicate air pollution to change public attitudes and behaviours? A review. Sustain Sci. (2021) 16:2027–47. doi: 10.1007/s11625-021-01038-2

[32] Marquart H Ueberham M Schlink U. Extending the dimensions of personal exposure assessment: a methodological discussion on perceived and measured noise and air pollution in traffic. J Transp Geogr. (2021) 93:103085. doi: 10.1016/j.jtrangeo.2021.103085

[33] Gössling S Humpe A Litman T Metzler D. Effects of perceived traffic risks, noise, and exhaust smells on bicyclist behaviour: an economic evaluation. Sustain For. (2019) 11:408. doi: 10.3390/su11020408

[34] Hou Y Gao M Huang L Wang Q. Air pollution reduces interpersonal trust: the roles of emotion and emotional susceptibility. Int J Environ Res Public Health. (2021) 18:5631. doi: 10.3390/ijerph18115631, PMID: 34070334 PMC8197547

[35] Elliott SJ Cole DC Krueger P Voorberg N Wakefield S. The power of perception: Health risk attributed to air pollution in an urban industrial Neighbourhood. Risk Anal. (1999) 19:621–34. doi: 10.1111/j.1539-6924.1999.tb00433.x 10765426

[36] Schmitz S Weiand L Becker S Niehoff N Schwartzbach F von Schneidemesser E. An assessment of perceptions of air quality surrounding the implementation of a traffic-reduction measure in a local urban environment. Sustain Cities Soc. (2018) 41:525–37. doi: 10.1016/j.scs.2018.06.011

[37] Egondi T Kyobutungi C Ng N Muindi K Oti S De VSV. Community perceptions of air pollution and related health risks in Nairobi slums. Int J Environ Res Public Health. (2013) 10:4851–68. doi: 10.3390/ijerph10104851, PMID: 24157509 PMC3823347

[38] Guo Y Liu F Lu Y Mao Z Lu H Wu Y. Factors affecting parent’s perception on air quality—from the individual to the community level. Int J Environ Res Public Health. (2016) 13:493. doi: 10.3390/ijerph13050493, PMID: 27187432 PMC4881118

[39] Maione M Mocca E Eisfeld K Kazepov Y Fuzzi S. Public perception of air pollution sources across Europe. Ambio. (2021) 50:1150–8. doi: 10.1007/s13280-020-01450-5, PMID: 33382442 PMC8068740

[40] Liao X Tu H Maddock JE Fan S Lan G Wu Y. Residents’ perception of air quality, pollution sources, and air pollution control in Nanchang, China. Atmos Pollut Res. (2015) 6:835–41. doi: 10.5094/APR.2015.092

[41] Orru K Nordin S Harzia H Orru H. The role of perceived air pollution and health risk perception in health symptoms and disease: a population-based study combined with modelled levels of PM10. Int Arch Occup Environ Health. (2018) 91:581–9. doi: 10.1007/s00420-018-1303-x, PMID: 29602966 PMC6002462

[42] Cesaroni G Badaloni C Porta D Forastiere F Perucci CA. Comparison between various indices of exposure to traffic-related air pollution and their impact on respiratory health in adults. Occup Environ Med. (2008) 65:683–90. doi: 10.1136/oem.2007.037846, PMID: 18203803 PMC2771851

[43] Oltra C Sala R. Communicating the risks of urban air pollution to the public. A study of urban air pollution information services. Rev Int Contam Ambient. (2015) 31:361–75.

[44] Zhu J Lu C Wei Z. Perception of air pollution and the evaluation of local governments’ environmental governance: an empirical study on China. Atmosfera. (2023) 14:212. doi: 10.3390/atmos14020212

[45] Reames TG Bravo MA. People, place and pollution: investigating relationships between air quality perceptions, health concerns, exposure, and individual- and area-level characteristics. Environ Int. (2019) 122:244–55. doi: 10.1016/j.envint.2018.11.013, PMID: 30449629

[46] Oltra C Sala R. Perception of risk from air pollution and reported behaviors: a cross-sectional survey study in four cities. J Risk Res. (2018) 21:869–84. doi: 10.1080/13669877.2016.1264446

[47] Ramírez AS Ramondt S Van Bogart K Perez-Zuniga R. Public awareness of air pollution and Health threats: challenges and opportunities for communication strategies to improve environmental Health literacy. J Health Commun. (2019) 24:75–83. doi: 10.1080/10810730.2019.1574320, PMID: 30730281 PMC6688599

[48] Siddique AB Sujan MSH Ahmed S Ishadi KS Tasnim R Islam MS. Assessment of knowledge, attitudes, and practice regarding air pollution and health effects among general people: a multi-divisional cross-sectional study in Bangladesh. PLoS One. (2024) 19:e0305075. doi: 10.1371/journal.pone.0305075, PMID: 38861559 PMC11166284

[49] Idler EL Benyamini Y. Self-rated health and mortality: a review of twenty-seven community studies. J Health Soc Behav. (1997) 38:21–37. doi: 10.2307/2955359, PMID: 9097506

[50] Ou JY Peters JL Levy JI Bongiovanni R Rossini A Scammell MK. Self-rated health and its association with perceived environmental hazards, the social environment, and cultural stressors in an environmental justice population. BMC Public Health. (2018) 18:970. doi: 10.1186/s12889-018-5797-7, PMID: 30075713 PMC6090753

[51] Deng Q Zhao J Liu W Li Y. Heatstroke at home: prediction by thermoregulation modeling. Build Environ. (2018) 137:147–56. doi: 10.1016/j.buildenv.2018.04.017

[52] Grigorieva E Lukyanets A. Combined effect of hot weather and outdoor air pollution on respiratory Health: literature review. Atmosfera. (2021) 12:790. doi: 10.3390/atmos12060790

[53] Zhao J Wang Y Ou D Wang H Li Y Deng Q. Predicting survival time for cold exposure by thermoregulation modeling. Build Environ. (2024) 249:111127. doi: 10.1016/j.buildenv.2023.111127

[54] Lee B-J Kim B Lee K. Air pollution exposure and cardiovascular disease. Toxicol Res. (2014) 30:71–5. doi: 10.5487/TR.2014.30.2.071, PMID: 25071915 PMC4112067

[55] WHO (2023). Environment and health EURO. Available online at: https://www.who.int/europe/health-topics/environmental-health (Accessed August 22, 2023)

[56] Al-Kindi SG Brook RD Biswal S Rajagopalan S. Environmental determinants of cardiovascular disease: lessons learned from air pollution. Nat Rev Cardiol. (2020) 17:656–72. doi: 10.1038/s41569-020-0371-2, PMID: 32382149 PMC7492399

[57] Cohen AJ Ross Anderson H Ostro B Pandey KD Krzyzanowski M Künzli N. The global burden of disease due to outdoor air pollution. J Toxicol Environ Health A. (2005) 68:1301–7. doi: 10.1080/15287390590936166, PMID: 16024504

[58] EEA. (2023). Ηow air pollution affects our health. Available online at: https://www.eea.europa.eu/en/topics/in-depth/air-pollution/eow-it-affects-our-health (Accessed August 22, 2023)

[59] Chowdhury Z Le LT Masud AA Chang KC Alauddin M Hossain M. Quantification of indoor air pollution from using Cookstoves and estimation of its health effects on adult women in Northwest Bangladesh. Aerosol Air Qual Res. (2012) 12:463–75. doi: 10.4209/aaqr.2011.10.0161

[60] Sarwar G Hogrefe C Henderson BH Foley K Mathur R Murphy B. Characterizing variations in ambient PM2.5 concentrations at the U.S. embassy in Dhaka, Bangladesh using observations and the CMAQ modeling system. Atmos Environ. (2023) 296:119587. doi: 10.1016/j.atmosenv.2023.119587, PMID: 37854171 PMC10581604

[61] Rahman MM Tasnim F Quader MA Bhuiyan MN-U-I Sakib MS Tabassum R. Perceived noise pollution and self-reported Health status among adult population of Bangladesh. Int J Environ Res Public Health. (2022) 19:2394. doi: 10.3390/ijerph19042394, PMID: 35206582 PMC8872462

[62] Krejcie RV Morgan DW. Determining sample size for research activities. Educ Psychol Meas. (1970) 30:607–10. doi: 10.1177/001316447003000308

[63] RStudio (n.d.). Open source & professional software for data science teams. Available online at: https://rstudio.com/ (Accessed January 15, 2021).

[64] Welcome to Python.org. (n.d.). Python.org. Available online at: https://www.python.org/ (Accessed January 15, 2021).

[65] Badland HM Duncan MJ. Perceptions of air pollution during the work-related commute by adults in Queensland, Australia. Atmos Environ. (2009) 43:5791–5. doi: 10.1016/j.atmosenv.2009.07.050

[66] Piro FN Madsen C Næss Ø Nafstad P Claussen B. A comparison of self reported air pollution problems and GIS-modeled levels of air pollution in people with and without chronic diseases. Environ Health. (2008) 7:9. doi: 10.1186/1476-069X-7-9, PMID: 18307757 PMC2289819

[67] Bano R Khayyam U. Industrial air pollution and self-reported respiratory and irritant health effects on adjacent residents: a case study of Islamabad industrial estate (IEI). Air Qual Atmos Health. (2021) 14:1709–22. doi: 10.1007/s11869-021-01051-5

[68] Al Ahad MA Demšar U Sullivan F Kulu H. Does long-term air pollution exposure affect self-reported Health and limiting long term illness disproportionately for ethnic minorities in the UK? A census-based individual level analysis. Appl Spat Anal Policy. (2022) 15:1557–82. doi: 10.1007/s12061-022-09471-1

[69] Abed Al Ahad M Demšar U Sullivan F Kulu H. The spatial–temporal effect of air pollution on individuals’ reported health and its variation by ethnic groups in the United Kingdom: a multilevel longitudinal analysis. BMC Public Health. (2023) 23:897. doi: 10.1186/s12889-023-15853-y, PMID: 37189130 PMC10186783

[70] Health I on U. (2020). Air pollution and older people – reports. Impact Urban Health. Available online at: https://urbanhealth.org.uk/insights/reports/air-pollution-and-older-people (Accessed August 27, 2023)

[71] Simoni M Baldacci S Maio S Cerrai S Sarno G Viegi G. Adverse effects of outdoor pollution in the elderly. J Thorac Dis. (2015) 7:34–45. doi: 10.3978/j.issn.2072-1439.2014.12.10, PMID: 25694816 PMC4311079

[72] Andrade A D’Oliveira A De Souza LC ACR B De F Dominski FH. Effects of air pollution on the Health of older adults during physical activities: mapping review. Int J Environ Res Public Health. (2023) 20:3506. doi: 10.3390/ijerph20043506, PMID: 36834200 PMC9960154

[73] Poland GA Ovsyannikova IG Kennedy RB Lambert ND Kirkland JL. A systems biology approach to the effect of aging, immunosenescence and vaccine response. Curr Opin Immunol. (2014) 29:62–8. doi: 10.1016/j.coi.2014.04.005, PMID: 24820347 PMC4119552

[74] Jung C Alqassimi N El Samanoudy G. The comparative analysis of the indoor air pollutants in occupied apartments at residential area and industrial area in Dubai, United Arab Emirates. Front Built Environ. (2022) 8:998858. doi: 10.3389/fbuil.2022.998858, PMID: 40061943

[75] Alharbi BH Alhazmi HA Aldhafeeri ZM. Air quality of work, residential, and traffic areas during the COVID-19 lockdown with insights to improve air quality. Int J Environ Res Public Health. (2022) 19:727. doi: 10.3390/ijerph19020727, PMID: 35055549 PMC8775798

[76] Zahra SI Iqbal MJ Ashraf S Aslam A Ibrahim M Yamin M. Comparison of ambient air quality among industrial and residential areas of a typical south Asian City. Atmosfera. (2022) 13:1168. doi: 10.3390/atmos13081168

[77] Joseph J Patil RS Gupta SK. Estimation of air pollutant emission loads from construction and operational activities of a port and harbour in Mumbai, India. Environ Monit Assess. (2009) 159:85–98. doi: 10.1007/s10661-008-0614-x, PMID: 19030999

[78] Huang K Zhuang G Lin Y Wang Q Fu JS Fu Q. How to improve the air quality over megacities in China: pollution characterization and source analysis in Shanghai before, during, and after the 2010 world expo. Atmos Chem Phys. (2013) 13:5927–42. doi: 10.5194/acp-13-5927-2013

[79] Mannan M Al-Ghamdi SG. Indoor air quality in buildings: a comprehensive review on the factors influencing air pollution in residential and commercial structure. Int J Environ Res Public Health. (2021) 18:3276. doi: 10.3390/ijerph18063276, PMID: 33810001 PMC8004912

[80] de Moraes RJB Costa DB Araújo IPS. Particulate matter concentration from construction sites: concrete and masonry works. J Environ Eng. (2016) 142:05016004. doi: 10.1061/(ASCE)EE.1943-7870.0001136

[81] Wieser AA Scherz M Passer A Kreiner H. Challenges of a healthy built environment: air pollution in construction industry. Sustain For. (2021) 13:10469. doi: 10.3390/su131810469

[82] Sekhavati E Yengejeh RJ. Particulate matter exposure in construction sites is associated with health effects in workers. Front Public Health. (2023) 11:1130620. doi: 10.3389/fpubh.2023.1130620, PMID: 36960377 PMC10028260

[83] Tribune Dhaka. (2020). Study: construction the leading cause of outdoor pollution. Dhaka Trib. Available online at: https://www.dhakatribune.com/science-technology-environment/environment/234522/study-construction-the-leading-cause-of-outdoor (Accessed August 27, 2023).

[84] Zuo J Rameezdeen R Hagger M Zhou Z Ding Z. Dust pollution control on construction sites: awareness and self-responsibility of managers. J Clean Prod. (2017) 166:312–20. doi: 10.1016/j.jclepro.2017.08.027

[85] Tao G Feng J Feng H Feng H Zhang K. Reducing construction dust pollution by planning construction site layout. Buildings. (2022) 12:531. doi: 10.3390/buildings12050531

[86] Sultana F. (2022). Development projects and air pollution. Available online at: https://bangladeshpost.net/posts/development-projects-and-air-pollution-79830 (Accessed August 27, 2023).

[87] Mahmud I. (2022). Air quality worst due to neglect by govt agencies. Prothomalo. Available online at: https://en.prothomalo.com/environment/pollution/air-quality-worst-due-to-neglect-by-govt-agencies (Accessed August 27, 2023).

[88] Groskop V. (2023). My Covid-era confidence crisis: how to regain your sense of self, hope and happiness. The Guardian. Available online at: https://www.theguardian.com/lifeandstyle/2023/may/31/my-covid-confidence-crisis-how-to-regain-your-sense-of-self-health-and-happiness (Accessed August 28, 2023).

[89] Bedrov A Bulaj G. Improving self-esteem with motivational quotes: opportunities for digital Health Technologies for People with Chronic Disorders. Front Psychol. (2018) 9:2126. doi: 10.3389/fpsyg.2018.02126, PMID: 30450071 PMC6224439

[90] BreatheLife. (2023) BreatheLife – a global campaign for clean air. BreatheLife2030. Available online at: https://breathelife2030.org/ (Accessed August 28, 2023)

[91] Action for Clean Air. (2023). Clean air day. Action Clean Air. Available online at: http://www.actionforcleanair.org.uk/campaigns/clean-air-day (Accessed August 28, 2023)

[92] Manisalidis I Stavropoulou E Stavropoulos A Bezirtzoglou E. Environmental and Health impacts of air pollution: a review. Front Public Health. (2020) 8:14. doi: 10.3389/fpubh.2020.00014, PMID: 32154200 PMC7044178

[93] Kodros JK O’Dell K Samet JM L’Orange C Pierce JR Volckens J. Quantifying the Health benefits of face masks and respirators to mitigate exposure to severe air pollution. GeoHealth. (2021) 5:e2021GH000482. doi: 10.1029/2021GH000482, PMID: 34541439 PMC8438762

[94] Tultrairatana S Phansuea P. Symptoms related to air pollution, mask-wearing and associated factors: a cross-sectional study among OPD pollution clinic patients in Bangkok, Thailand. J Health Res. (2021) 36:1058–67. doi: 10.1108/JHR-11-2020-0548, PMID: 35579975

[95] Rana MM. (2023). Will the new air pollution control rules save us? Dly Star. Available online at: https://www.thedailystar.net/opinion/views/news/will-the-new-air-pollution-control-rules-save-us-3238271 (Accessed August 28, 2023).

[96] Rahman MS Karamehic-Muratovic A Baghbanzadeh M Amrin M Zafar S Rahman NN. Climate change and dengue fever knowledge, attitudes and practices in Bangladesh: a social media–based cross-sectional survey. Trans R Soc Trop Med Hyg. (2020) 115:85–93. doi: 10.1093/trstmh/traa093, PMID: 32930796

[97] Abir T Ekwudu O Kalimullah NA Yazdani DMN-A Mamun AA Basak P. Dengue in Dhaka, Bangladesh: hospital-based cross-sectional KAP assessment at Dhaka north and Dhaka South City corporation area. PLoS One. (2021) 16:e0249135. doi: 10.1371/journal.pone.0249135, PMID: 33784366 PMC8009423

[98] Wang W Wu F Zhang F. Environmental city-regionalism in China: war against air pollution in the Beijing-Tianjin-Hebei region. Trans Plan Urban Res. (2023) 2:132–48. doi: 10.1177/27541223221144595

[99] Barwise Y Kumar P. Designing vegetation barriers for urban air pollution abatement: a practical review for appropriate plant species selection. Npj Clim Atmos Sci. (2020) 3:1–19. doi: 10.1038/s41612-020-0115-3, PMID: 40034341

[100] Diener A Mudu P. How can vegetation protect us from air pollution? A critical review on green spaces’ mitigation abilities for air-borne particles from a public health perspective - with implications for urban planning. Sci Total Environ. (2021) 796:148605. doi: 10.1016/j.scitotenv.2021.148605, PMID: 34271387

