# Supplementary material for: Perceived-air pollution and self-reported health status: a study on air pollution-prone urban area of Bangladesh
Source: Front Public Health. 2025 Apr 3;13:1382471. doi: 10.3389/fpubh.2025.1382471 (PMC12003290; doi:10.3389/fpubh.2025.1382471)
Supplement: Supplementary file 1 [file Table_1.docx]

**Perceived-Air Pollution and Self-reported Health Status: A Study on Air Pollution-prone Urban Area of Bangladesh**

**Appendix**

**TABLE 1.** Association between general air pollution-related health issues and perceived local air pollution (*n*=398).

| Features | Overall Air Pollution-Related Health Problem | | | |
| --- | --- | --- | --- | --- |
|  | *n* (%) | R^2^ | B^#^ | p-value |
| *1. Did the local air pollution of your area ever catch your attention?* | | | | |
| - No | 39 (9.80) | 0.002 | Reference |  |
| - Yes | 359 (90.20) |  | -0.02 | 0.296 |
| *2. Major sources of air pollution in your area* | | | | |
| - Burning of fossil fuel | 18 (4.52) | 0.089 | 0.08* | 0.010 |
| - Construction activities | 125 (31.41) |  | 0.08** | 0.005 |
| - Industrial emission | 19 (4.77) |  | -0.00 | 0.959 |
| - Motor vehicle emission | 27 (6.78) |  | -0.00 | 0.998 |
| - Openly burning waste | 29 (7.29) |  | 0.01 | 0.537 |
| - Combination of different sources (but not all of the above) | 169 (42.46) |  | -0.04** | 0.001 |
| - All of the above | 119 (29.90) |  | Reference |  |
| *3. Level of air pollution in the area can be controlled* | | | | |
| - Maybe | 102 (25.63) | 0.022 | Reference |  |
| - No | 44 (11.06) |  | 0.04* | 0.047 |
| - Yes | 252 (63.32) |  | -0.01 | 0.302 |
| *4. Which season has least air pollution?* | | | | |
| - Monsoon | 226 (56.78) | 0.017 | Reference |  |
| - Winter | 172 (43.22) |  | 0.03** | 0.009 |
| *5. Do you think indoor air pollution exists?* | | | | |
| - Maybe | 117 (29.40) | 0.007 | Reference |  |
| - No | 81 (20.35) |  | 0.01 | 0.536 |
| - Yes | 200 (50.25) |  | -0.01 | 0.313 |

^#^Beta; **p<0.05; **p<0.01.*

**TABLE 2.** Association between perceptions of air pollution (including its effects and mitigation efforts) and the total health problem caused by air pollution (*n*=398).

| Features | Overall Air Pollution-Related Health Problem | | | |
| --- | --- | --- | --- | --- |
|  | ***n* (%)** | **R^2^** | **B^#^** | **p-value** |
| *1. Air pollution is responsible for diseases among family or friends* | | | | |
| - Maybe | 110 (27.64) | 0.000 | Reference |  |
| - No | 69 (17.34) |  | -0.00 | 0.833 |
| - Yes | 219 (55.03) |  | 0.00 | 0.852 |
| *2. Air pollution is harmful for my health* | | | | |
| - Maybe | 28 (7.04) | 0.010 | Reference |  |
| - No | 27 (6.78) |  | 0.00 | 0.817 |
| - Yes | 343 (86.18) |  | -0.03 | 0.174 |
| *3. Are you aware of any air pollution-related campaign?* | | | | |
| - Maybe | 51 (12.81) | 0.038 | Reference |  |
| - No | 241 (60.55) |  | -0.02 | 0.261 |
| - Yes | 106 (26.63) |  | 0.03 | 0.088 |
| *4. Will you willingly participate in any air pollution-related awareness campaign?* | | | | |
| - Maybe | 130 (32.66) | 0.034 | Reference |  |
| - No | 54 (13.57) |  | 0.06** | 0.001 |
| - Yes | 214 (53.77) |  | -0.00 | 0.794 |
| *5. Air pollution-related information should be more available to the general people* | | | | |
| - Maybe | 44 (11.06) | 0.019 | Reference |  |
| - No | 13 (3.27) |  | 0.10** | 0.007 |
| - Yes | 341 (85.68) |  | 0.03 | 0.069 |
| *6. Only government and non-government organizations are responsible for controlling air pollution* | | | | |
| - Maybe | 44 (11.06) | 0.058 | Reference |  |
| - No | 234 (58.79) |  | -0.043* | 0.027 |
| - Yes | 120 (30.15) |  | 0.021 | 0.317 |
| *7. Friends and family members can play a role to reduce air pollution* | | | | |
| - Maybe | 62 (15.58) | 0.069 | Reference |  |
| - No | 44 (11.06) |  | 0.09*** | 0.000 |
| - Yes | 292 (73.37) |  | -0.00 | 0.588 |
| *8. Do you believe taking protective measures against air pollution may reduce health risk?* | | | | |
| - Maybe | 35 (8.79) | 0.018 | Reference |  |
| - No | 44 (11.06) |  | -0.00 | 0.865 |
| - Yes | 319 (80.15) |  | -0.04* | 0.044 |
| *9. Are you agree to pay increased tax and extra money to reduce air pollution?* | | | | |
| - Maybe | 103 (25.88) | 0.001 | Reference |  |
| - No | 100 (25.13) |  | 0.00 | 0.893 |
| - Yes | 195 (48.99) |  | 0.00 | 0.544 |
| *10. Follow daily weather forecast before going outside* | | | | |
| - No | 217 (54.52) | 0.000 | Reference |  |
| - Yes | 181 (45.48) |  | 0.00 | 0.537 |
| *11. Do you wear mask when you go outside?* | | | | |
| - No | 77 (19.35) | 0.020 | Reference |  |
| - Sometimes | 133 (33.42) |  | -0.04* | 0.023 |
| - Yes | 188 (47.24) |  | -0.04** | 0.006 |

^#^Beta; **p<0.05; **p<0.01; ***p<0.001.*
